# Bouveret’s Syndrome: A Rare Form of Gallstone Ileus Caused by Large Renal Cysts

**DOI:** 10.7759/cureus.39991

**Published:** 2023-06-05

**Authors:** Milos Kňazovický, Tomáš Gajdzik, Kleanthia Efthymiou, Veronika Roškovičová, Peter Závacký, René Hako, Martina Závacká, Jozef Radoňak

**Affiliations:** 1 1st Department of Surgery, Louis Pasteur University Hospital, Košice, SVK; 2 Clinic of Radiodiagnostics and Imaging Methods, Louis Pasteur University Hospital, Košice, SVK; 3 Clinic of Vascular Surgery, VUSCH (Východoslovenský ústav srdcových a cievnych chorôb, a.s., Košice, SVK

**Keywords:** difficult cholecystectomy, renal cysts, gallstone ileus, gastric outlet obstruction, bouveret syndrome

## Abstract

Bouveret’s syndrome is a rare variant of gallstone ileus caused by gastric outlet obstruction that arises from gallstones impacted in the distal stomach or proximal duodenum after passing through a cholecystoduodenal or cholecystogastric fistula. Simple kidney cysts are one of the most common lesions found in the elderly. They are usually asymptomatic, but the cysts can put pressure on the surrounding organs if they grow to large dimensions.This case report highlights a rare case of Bouveret’s syndrome due to the presence of a large gallstone in the pyloric region that was caused by the creation of a cholecystogastric fistula from pressure exerted by two giant cysts of the right kidney.

## Introduction

Bouveret’s syndrome is a rare variant of gallstone ileus, which is characterized by gastric outlet obstruction by a gallstone impacted either in the duodenum or stomach because of a cholecystoduodenal or cholecystogastric fistula. The syndrome was first published by French physician Leon Bouveret in 1896. It is a very rare complication of symptomatic cholelithiasis (0.3% to 0.5% of patients with gallstones) [1] and is associated with high rates of morbidity (60%) and mortality (12-30%) because it frequently occurs in older people with multiple comorbidities [2].

Simple renal cysts are one of the most common lesions found in the elderly. They are benign masses that, for unknown reasons, form in the kidneys and are usually detected incidentally either by ultrasound or CT scan [3]. Cysts that are smaller than 5 cm are usually asymptomatic and do not cause renal dysfunction. However, if they grow into a larger size, pressure against the surrounding organs in the abdominal cavity can lead to severe symptoms [4]. Simple kidney cysts causing gastrointestinal obstruction are quite rare, with only isolated cases recorded to date where large cysts exerted pressure on the gastrointestinal tract (GIT). No similar case reports have been published on bile duct compression or concomitant Bouveret’s syndrome. This case report highlights a rare case of Bouveret’s syndrome due to the presence of a large gallstone in the pyloric region that was caused by the creation of a cholecystogastric fistula from pressure exerted by two giant cysts of the right kidney.

## Case presentation

A 71-year-old man was admitted to the university hospital for further diagnostic procedures and surgical treatment after a four-day hospitalization in the surgical department of the district hospital. The first symptoms were loss of appetite, abdominal spasms, and subfebrilities, which the patient had reported for six months prior to presentation. These complaints gradually subsided. He had been recently treated only for systematic hypertension, while six years ago he recovered from septic bronchopneumonia. The first abdominal complaints were reported by the patient eight years ago when there was abdominal pain in the right hypochondrium with febrilities. An ultrasound of the abdomen revealed one gallstone approximately 1 cm in diameter and small cysts on the right kidney. At that time, the pain and fever gradually subsided after the administration of anti-inflammatory drugs.

**Figure 1:**
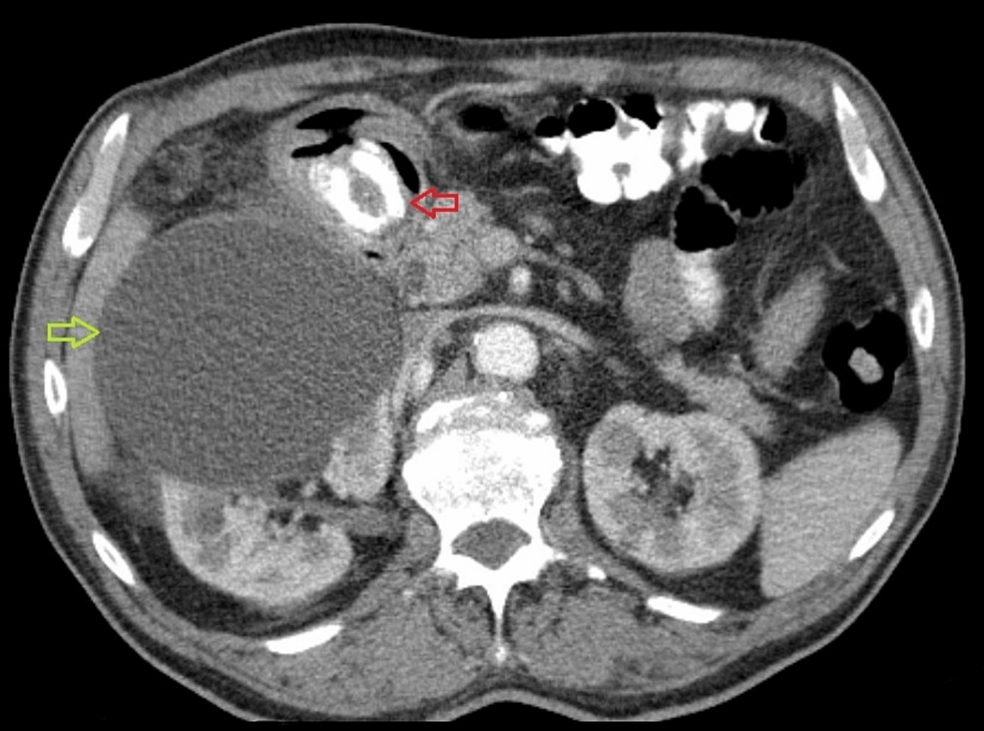
Abdominal CT (axial scan) Gallstone in the pyloric area of the stomach (red arrow) and large right kidney cyst (yellow arrow) can be seen.

**Figure 2:**
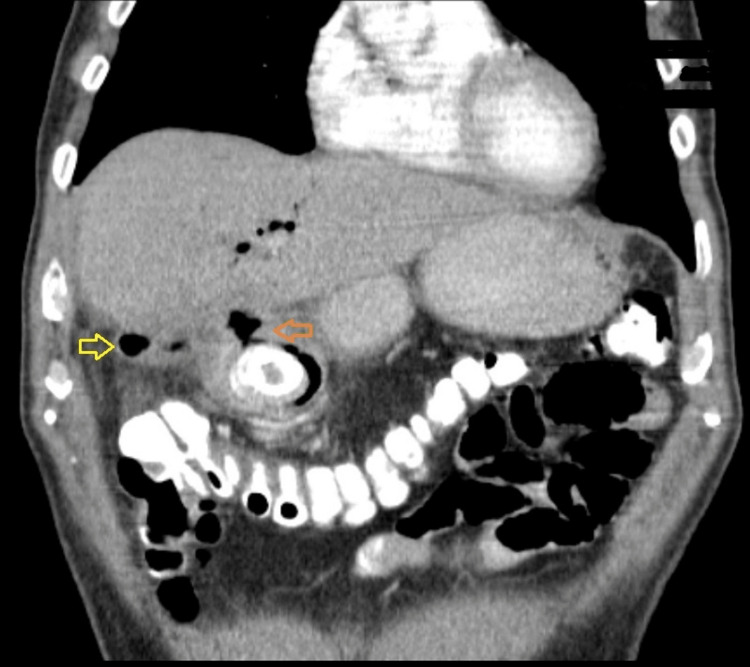
Abdominal CT (coronary reconstruction) Contracted cholecyst (yellow arrow), cholecystogastric fistula (orange arrow), and signs of pneumobilia in the liver parenchyma can be seen.

**Figure 3:**
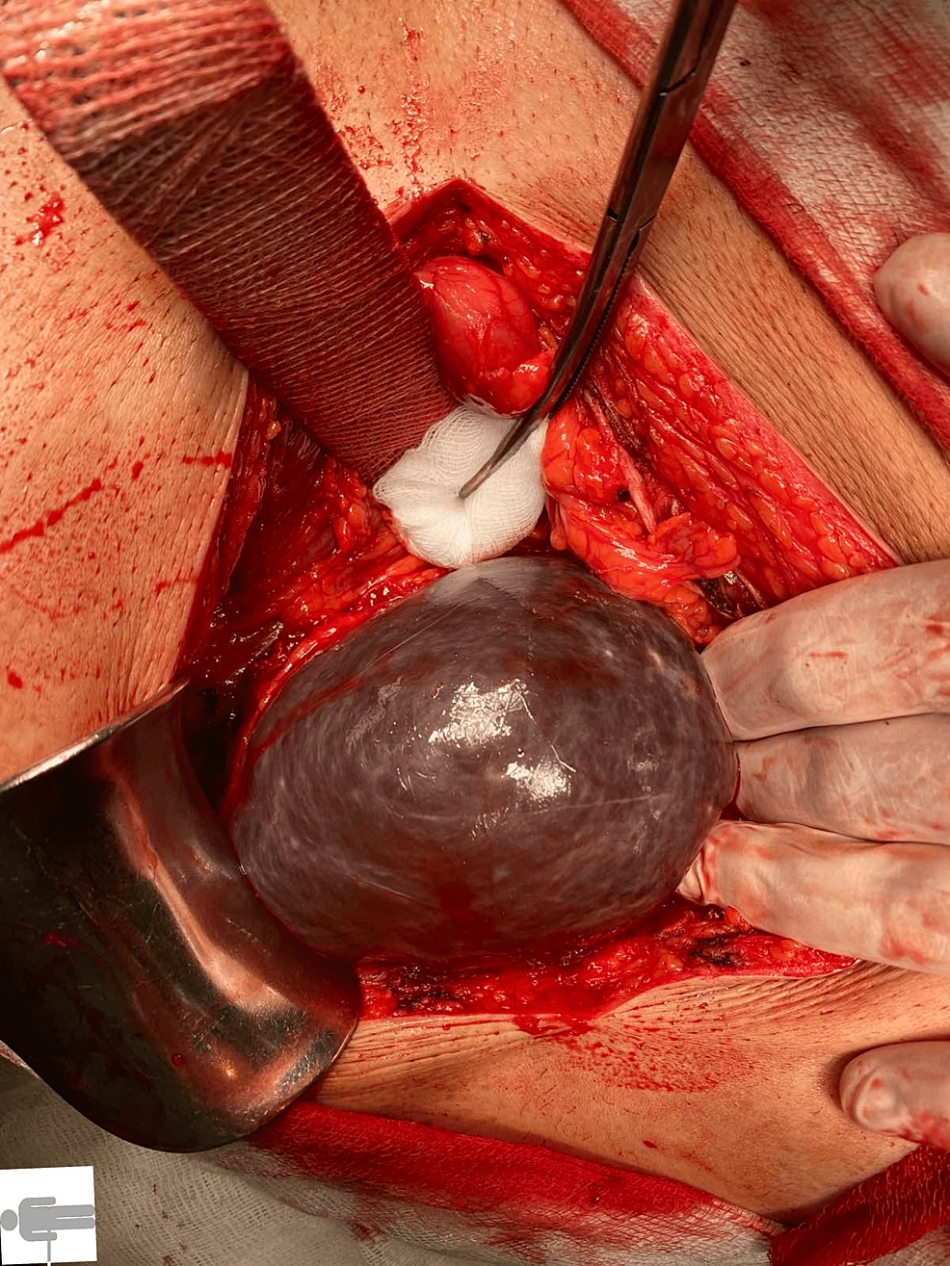
A large right kidney cyst

**Figure 4:**
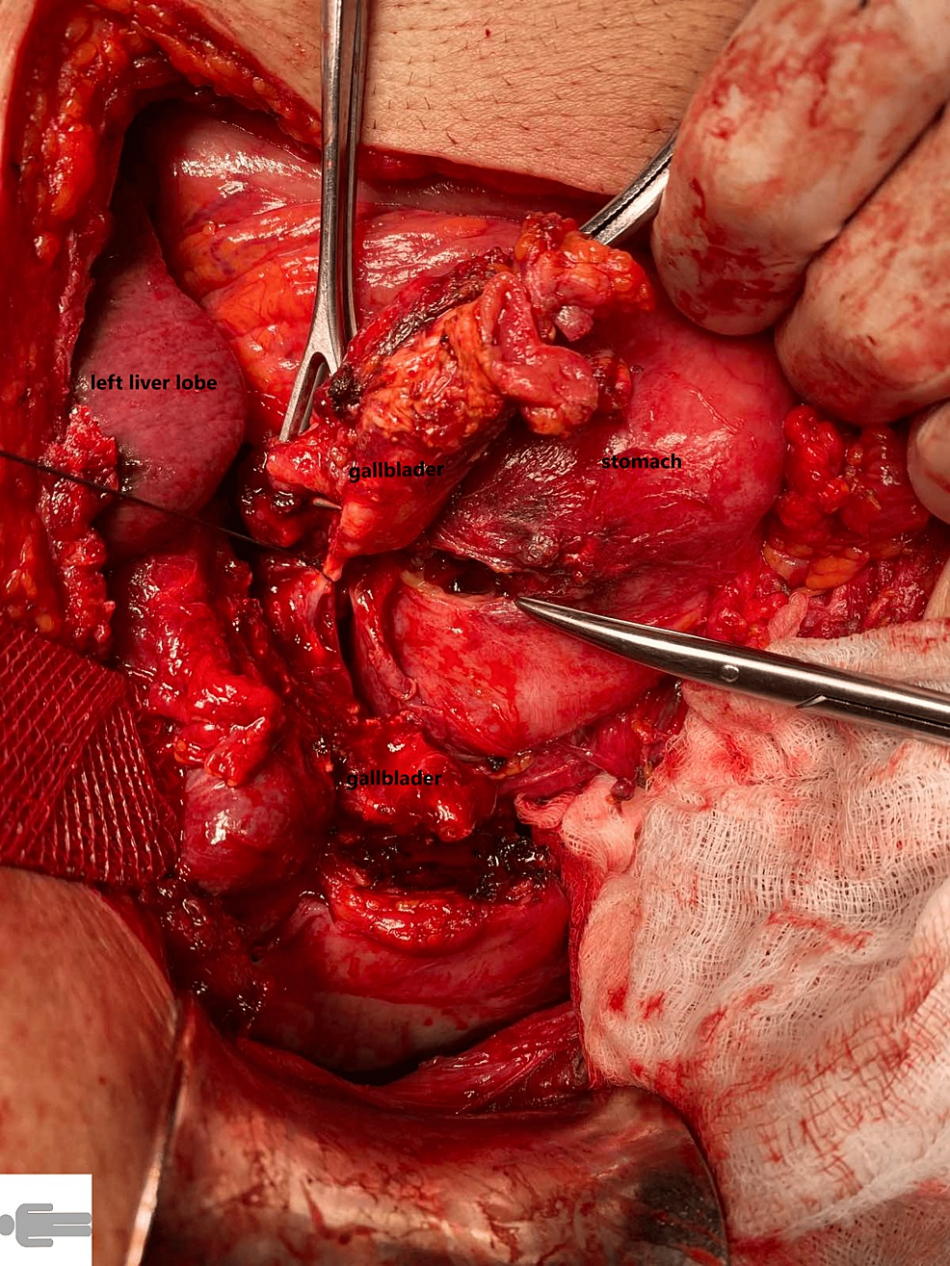
Cholecystogastric fistula

**Figure 5:**
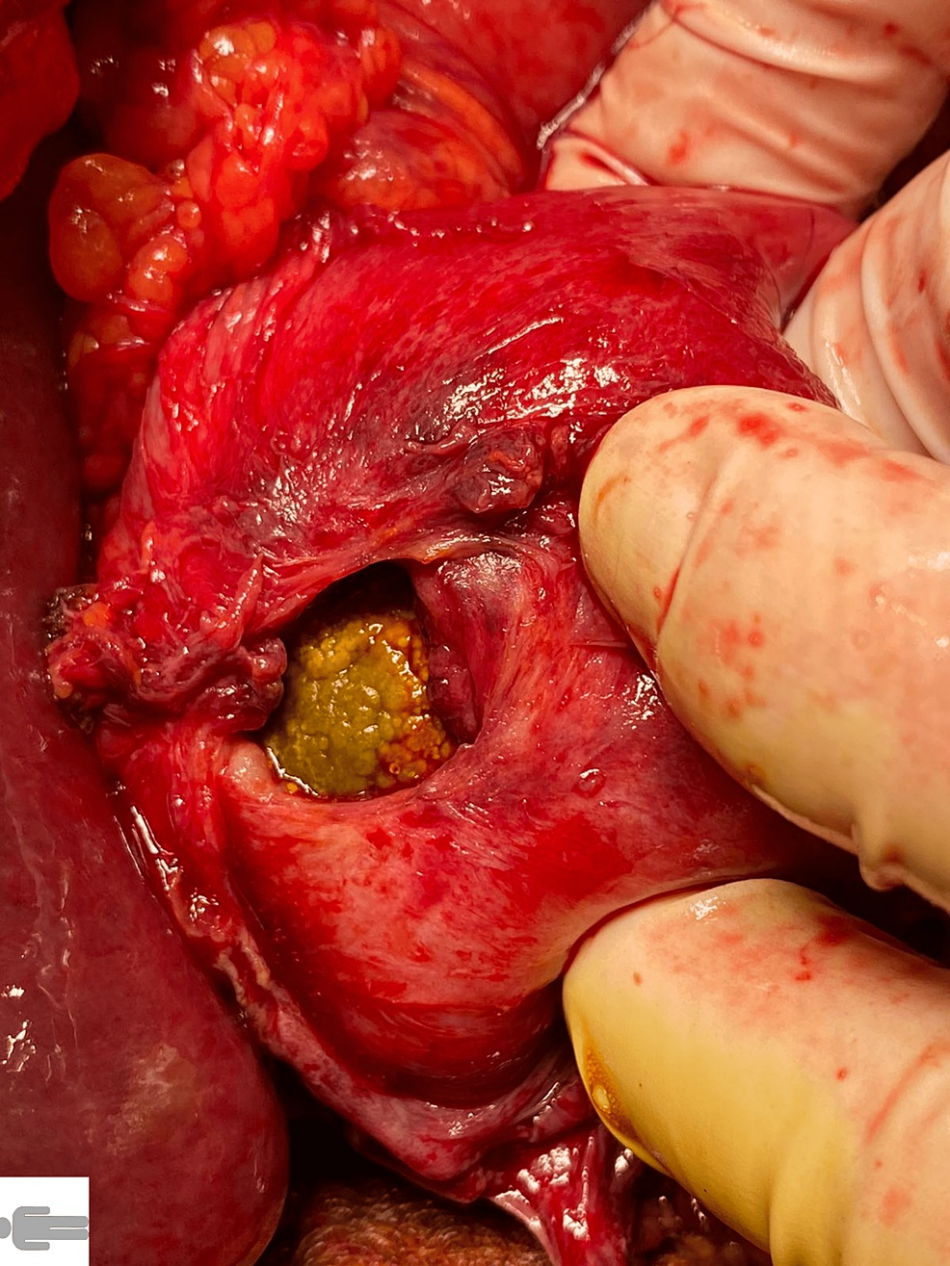
Gallstone in the pyloric area

**Figure 6:**
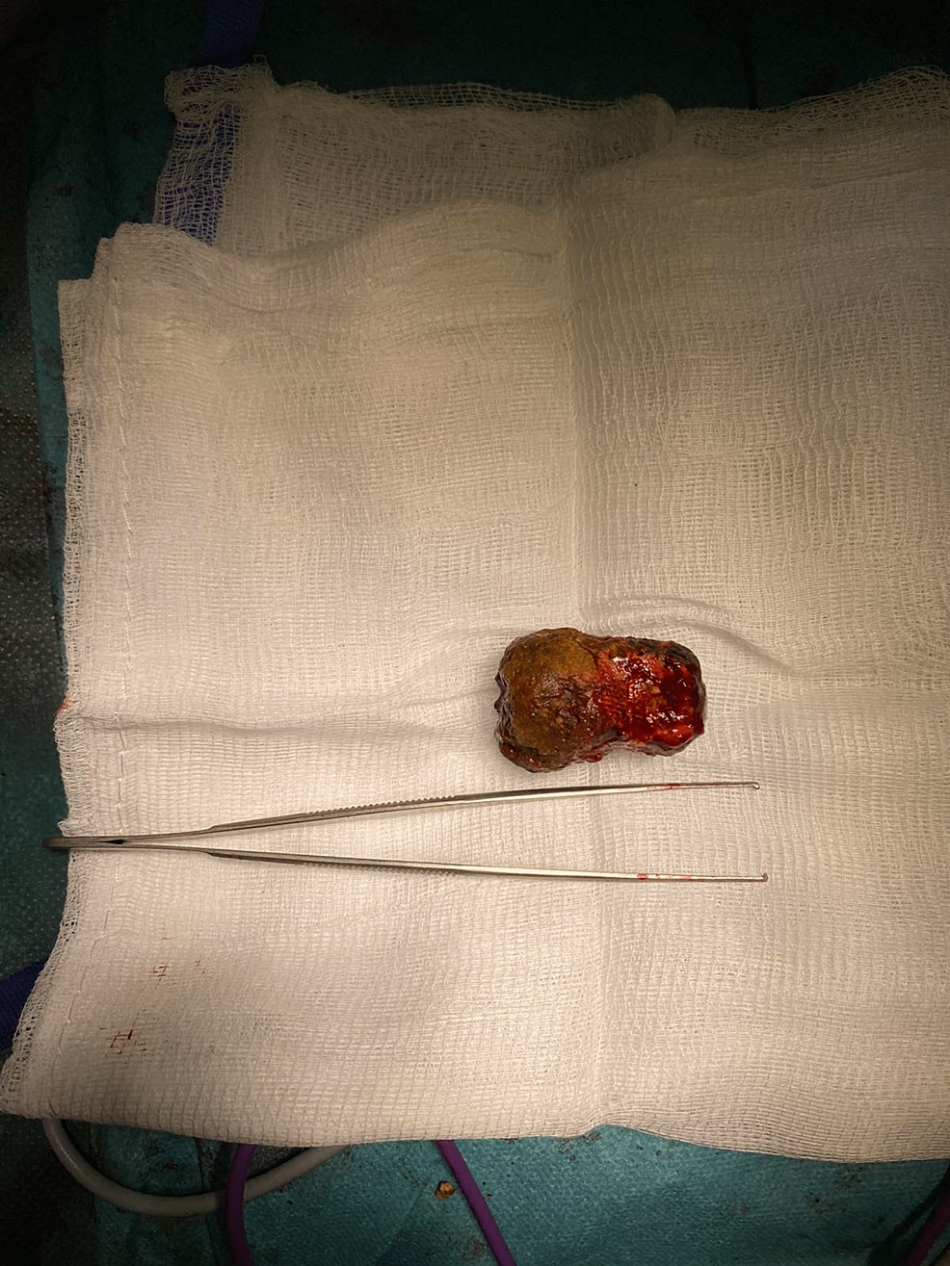
Extracted gallstone (forceps is 16 cm long)

At the time of admission, the patient felt epigastric pain with recurrent vomiting. Both nausea and vomiting occurred shortly after the intake of food, which he had only been able to consume in liquid form over the previous few days. Upon admission, the patient had no fever, his pulse was 70 beats per minute, and there were no cardiac rhythm disturbances or jaundice. Palpation during physical examination revealed tenderness in the upper central region of the abdomen. Initial labs showed a white blood cell count of 13.9 x 109/L and a C-reactive protein level of 24 mg/L. All other laboratory results, such as hemoglobin, bilirubin, and renal tests, were normal. An abdominal ultrasound examination at the district hospital concluded that neither cholecystolithiasis nor distension of the stomach could explain the ileus; therefore, the patient subsequently underwent a CT scan of the abdomen. It found two large subcapsular cysts from the right kidney, measuring 12 x 13 x 22 cm (Figure 1), both of which had drifted deeper into the liver parenchyma and were partially compressing the inferior vena cava. More extensive parenchymal and subcapsular cysts were also seen on the left kidney. Extensive presence of gas, along with large amounts of air, was also detected in the common bile duct. Pressure from the cysts had caused the gallbladder to displace to the wall of the stomach, with subsequent fistulation between the stomach and gallbladder. The area around the gallbladder was distended with air bubbles, and an abscess was developing. On the initial CT scan, the gallstone was incorrectly described in the gallbladder (Figure 2), and gastroscopy was eventually abandoned. The urologist who was consulted did not recommend urgent urological surgery. The patient received a nasogastric tube, followed by infusion of spasmolytics and treatment with antibiotics, which gradually eased the discomfort. Cholecystectomy at the local hospital was rejected by the patient, and he was discharged at his own request. The patient was subsequently admitted to the surgical clinic of the university hospital, where he underwent surgery the next day. A laparotomy was performed with a subcostal incision, where the subhepatic area was not visible during revision due to the presence of inflammatory infiltrate from the gallbladder, stomach, and duodenum. The inflammatory mass was compressed inferiorly and laterally by two giant cysts of the right kidney, 20 cm in diameter in the craniocaudal direction, which had drifted into the parenchyma of the liver (Figure 3). After loosening adhesions in the area of the inflammatory infiltrate, both of the right kidney cysts were extirpated, with a sample of 3,000 mL of serous fluid sent for cultivation and cytological screening. Afterward, the contracted gallbladder was removed through a combined retrograde and antegrade cholecystectomy. An opening measuring 2.5 cm in diameter was found in the pyloric region at the site of the cholecystogastric fistula (Figure 4). After duodenal mobilization, a gallstone 6 x 3 x 3 cm was found in the pyloric canal (Figure 5) causing ventilating obstruction (Figure 6). The stone was extracted through the fistula, followed by excision of the fistula walls and fistula suture in two layers on an inserted nasogastric tube with subsequent omentoplasty. A Tygon drain was then inserted subhepatically, and the abdominal cavity was closed in anatomical layers.

There were no postoperative complications. Nasogastric tube and abdominal drain were removed six days after the operation. The patient was gradually refed, the pathological values of the laboratory parameters gradually normalized, and the surgical wound healed primarily. Ten days after surgery, the patient was discharged from the hospital. Both the fluid culture and cytological screenings of the fluid from the right kidney cysts were negative for the presence of bacteria and malignant cells. Histopathological examination of the gallbladder showed chronic cholecystitis focally with evidence of pyloric metaplasia. Histopathological examination of the wall of the right renal cyst showed a simple renal cyst with areas of renal parenchyma with interstitial chronic inflammatory cellularization.

## Discussion

A diagnosis of Bouveret’s syndrome is very rare, affecting less than 0.5% of patients with gallstones [5]. Mechanical obstruction of the stomach or duodenum is caused by a larger-sized gallstone that travels out of the gallbladder through a cholecystogastric fistula or, more commonly, a cholecystoduodenal fistula [6]. Chronic inflammation of the gallbladder and surrounding structures forms adhesions between the biliary system and the intestines. A combination of inflammation and gallstones causes increasing intraluminal pressure, mucosal erosion, and ischemia of the gallbladder wall. The result is necrosis and perforation of the gallbladder wall, leading to the formation of a fistula between the gallbladder and the adjacent part of the GIT [7]. It is very rare for fistulae to form in patients with cholelithiasis, occurring in only 0.3% to 0.5% of cases overall. In the majority of cases, they are cholecystoduodenal (60%), cholecystocolic (17%), cholecystogastric (5%), or choledochoduodenal (5%) [8]. Impacted gallstones can range from 2 to 10 cm, with a median size of 4.3 cm [9]. When the diameter of cholecystolithiasis is greater than 2.5 cm, surgery is the most frequently adopted and the most effective treatment in such cases [10]. The most common site of obstruction is the ileocecal valve in the terminal ileum (70%), followed by the proximal jejunum and ileum [11]. Obstruction is rarest in the pylorus and the bulb of the duodenum and is found only in 1% to 3% of patients with biliary ileus [12].

A diagnosis of Bouveret’s syndrome depends on the clinical picture, laboratory testing (elevated levels of direct bilirubin, gamma-glutamyl transferase, and alkaline phosphatase), and imaging methods. In a clinical manifestation of biliary ileus, a history of previous biliary symptoms may be noted in 27-80% of patients [13]. Bouveret’s syndrome normally appears clinically with symptoms of gastric outlet obstruction, such as epigastric pain and vomiting after eating. Nausea and vomiting were documented in 86% of cases, while abdominal pain and discomfort occurred in 71% of them [7]. Bleeding from the upper GIT may be the first symptom of it in 15% of patients [5,14]. Because most gallstones are radiolucent and also mainly due to extensive gas formation, the ability of X-rays and abdominal ultrasounds to diagnose the cause of gastric outlet obstruction is usually quite limited. CT scans provide the best imaging technique for diagnosing gallstones and Bouveret’s syndrome [15]. They provide a high level of sensitivity and specificity when assessing gallbladder wall thickness (for detecting acute or chronic cholecystitis) and examining gallbladder contents (air, residual gallstones) and presence of gallstones [15,16]. It is also the best imaging technique for capturing the Rigler’s triad, a combination of findings specific for gallbladder ileus consisting of small bowel obstruction, pneumobilia, and ectopic gallstones. In recent years, magnetic resonance cholangiopancreatography (MRCP) has been increasingly used to diagnose this syndrome, becoming an effective alternative to CT scans and abdominal X-ray because MRCP can differentiate between fluid and gallstones, it can also identify fistulae, and no oral contrast is required [16].

Selecting the right treatment for Bouveret’s syndrome remains controversial. Nonsurgical treatment methods include endoscopic extraction of gallstones with mechanical lithotripsy and extracorporeal shockwave lithotripsy. But a disadvantage of these procedures is their low success rate (less than 10%) [13,17]. Direct endoscopic removal raises the risk of stone impaction into the esophagus, while lithotripsy will break apart the stone, with the possibility of the fragments migrating and consequently obstructing the terminal ileum. Other risks include bleeding and perforation of the intestinal wall. Therefore, surgery is required as a definitive treatment in more than 90% of patients [18]. The optimal approach to take particularly depends on the patient’s overall condition and comorbidities, with careful decision-making about invasiveness and optimal treatment time. There is still ongoing discussion about simultaneously performing a cholecystectomy and removing fistulae. Combining enterolithotomy, cholecystectomy, and fistula repair in a single surgical procedure has the advantage of preventing subsequent biliary complications. This operation is more invasive, carries a higher risk, and is usually performed only in selected low-risk patients [19,20]. On the other hand, for elderly and high-risk patients with multiple comorbidities, two-stage surgery is ordinarily preferred, which involves extracting the obstructing stone from the gastrotomy or enterotomy and postponing cholecystectomy with fistula repair to later date [21].

Simple renal cysts are commonly found even in normal kidneys. They are mostly acquired lesions, whereas bilateral, multiple cysts and cysts found at a young age tend to have a genetic basis. They are present in 10% of the population, and their prevalence as well as the number and dimensions of cysts increase significantly with advancing age [3,22]. The average growth rate is 2.82 mm per year, while tending to be greater in younger patients and in patients with multiple cysts, in whom the rate rises up to 6.93 mm per year [23]. Most simple renal cysts are asymptomatic. However, there are rare cases in which they may be present with abdominal mass enlargement, rupture, hemorrhage, hematuria, infection, and hypertension or renal obstruction [24]. Simple cysts only need to be treated when their large dimensions cause clinical manifestations and complications. In this cases, treatment consists of needle aspiration with sclerotherapy under ultrasound control, or either laparoscopic or open surgical removal of the cyst wall.

This case presents a rare combination of cholecystolithiasis and large renal cysts, which are otherwise common diseases. In addition, a diagnosis of Bouveret’s syndrome tends to be more challenging because it usually comes late, thus contributing to the high mortality rate. Cases of simple renal cysts as a cause of gastrointestinal obstruction are likewise very rare, with only isolated cases recorded to date where large cysts exerted pressure on the GIT. Cases have been published of large left kidney cysts that have caused partial intestinal obstruction [25], chronic intestinal obstruction in the splenic flexure [26], and direct compression of the stomach [27]. There has also been a case of large cysts of the right kidney that caused obstruction of the duodenum [28]. No similar case reports have been published on bile duct compression or concomitant Bouveret’s syndrome. In this case, the patient was an elderly man with no chronic systemic disease who was not part of the population usually susceptible to Bouveret's syndrome. The clinical signs were week-long abdominal pain with recurrent vomiting, abdominal spasms, and subfebrile temperature. The patient’s case history and the CT scan suggested an unusual clinical situation with the development of an intermittent pyloric obstruction after a large stone had migrated through a cholecystogastric fistula. On the initial CT scan, it was misinterpreted as cholecystolithiasis with gallstone in the gallbladder. In addition, large subcapsular cysts were present on the right kidney, which had deeply intervened into the liver parenchyma and partially compressed the inferior vena cava. The pressure caused dislocation of the gallbladder to the wall of the stomach and subsequent fistulation between the stomach and gallbladder. Since the cysts were not septated and the walls not irregular, it was unlikely for them to be malignant [29]. Because endoscopic treatment methods tend to be unsuccessful for stones greater than 2.5 cm in size [30], the only possible solution was a surgical procedure that would simultaneously remove the two huge renal cysts so that they would no longer be pressing against the surrounding organs, and the gastric obstruction by extracting the large gallstone from the gastrotomy while seeking to avoid recurrence and other cholecystolithiasis complications by also performing a cholecystectomy.

## Conclusions

Bouveret’s syndrome is a rare cause of pyloric obstruction and is often misdiagnosed. A combination of an endoscopy and abdominal CT scan is the best method for discovering and diagnosing it. Unless the condition can be resolved with an endoscopy, surgery is indicated, the priority of which is to remove the obstruction in the stomach or duodenum, excluding distal obstruction. The simultaneous presence of two rare intra-abdominal pathologies may also become a diagnostic challenge. Larger-sized simple renal cysts should also be included in any differential diagnosis of upper GIT obstruction caused by external factors.
